# A Horizontal Alignment Tool for Numerical Trend Discovery in Sequence Data: Application to Protein Hydropathy

**DOI:** 10.1371/journal.pcbi.1003247

**Published:** 2013-10-10

**Authors:** Omar Hadzipasic, James O. Wrabl, Vincent J. Hilser

**Affiliations:** 1 Department of Biology, Johns Hopkins University, Baltimore, Maryland, United States of America; 2 T.C. Jenkins Department of Biophysics, Johns Hopkins University, Baltimore, Maryland, United States of America; Wake Forest University, United States of America

## Abstract

An algorithm is presented that returns the optimal pairwise gapped alignment of two sets of signed numerical sequence values. One distinguishing feature of this algorithm is a flexible comparison engine (based on both relative shape and absolute similarity measures) that does not rely on explicit gap penalties. Additionally, an empirical probability model is developed to estimate the significance of the returned alignment with respect to randomized data. The algorithm's utility for biological hypothesis formulation is demonstrated with test cases including database search and pairwise alignment of protein hydropathy. However, the algorithm and probability model could possibly be extended to accommodate other diverse types of protein or nucleic acid data, including positional thermodynamic stability and mRNA translation efficiency. The algorithm requires only numerical values as input and will readily compare data other than protein hydropathy. The tool is therefore expected to complement, rather than replace, existing sequence and structure based tools and may inform medical discovery, as exemplified by proposed similarity between a chlamydial ORFan protein and bacterial colicin pore-forming domain. The source code, documentation, and a basic web-server application are available.

## Author Summary

Trend discovery is an important way to generate understanding from large amounts of data. We have developed a novel tool that discovers significantly similar trends shared between two numerical data sets. Since the tool's algorithmic method compares both the relative shapes of the “peaks” and “valleys” in the data, as well as the absolute magnitudes of the numerical values, we believe the tool is tolerant of imperfections and could be applicable to a wide range of scientific, engineering, social, or economic problems. In short, if measurements can be converted to a series of numbers, our tool may potentially be useful for trend discovery. Since we are a protein biophysics group, we are most naturally interested in discovering new similarities between proteins, and we have discovered a particularly interesting, statistically significant similarity between a protein unique to *Chlamydia* and a bacterial pore-forming protein, colicin. This previously unreported similarity may have medical relevance, and we are currently experimentally testing the properties of the chlamydial protein in the laboratory. In a second example, we demonstrate the tool's ability to easily recover a known, but difficult to detect, relationship between two other GPCR proteins.

This is a *PLOS Computational Biology* Methods article.

## Introduction

Determining the evolutionary relatedness of two protein sequences is most successfully performed by amino acid sequence comparison [1]–[5]. However, it is well known that structure can be preserved even when sequence has diverged past the point of amino acid similarity recognition [6], suggesting that sequences can bestow local, subglobal, and global properties to a protein that can be preserved in the absence of strict conservation of the side chain atoms. In other words, similar properties could exist horizontally in a sequence even when recognizable vertical conservation is lost [7]. Even if such similarities are due to analogy rather than homology [8], approaches are needed that can augment sequence based analysis by matching patterns that may be independent of amino acid conservation at each position.

Comparison of three-dimensional atomic structures [9]–[13] is one example of such pattern matching. However, protein function and evolution arise from a manifold of physical, chemical, and biological mechanisms, only partly accounted for by side chain identity or structural similarity [14]–[18]. It may be the case that proteins can also be meaningfully characterized by other attributes, such as the energetic contributions to stability [19] or the predicted codon translation efficiency along the mRNA [20]–[22]. Yet, such attributes are not easily accommodated by simple adaptation of current algorithms, largely because the scoring systems for such algorithms are based on positional sequence identity (amino acid substitution matrices) or absolute geometric structural similarity (Euclidean distance).

As a result, properties other than sequence and structure, and their additional potential biological insight into proteins, have not been as thoroughly explored. For example, the local thermodynamic stability of a protein, as experimentally measured by deuterium-hydrogen exchange [23], [24], is described by a one-dimensional sequence of numerical values (*i.e.* amide protection factors). These values are well-known to be a combination of sequence, structure, and solvent effects [25], but no substitution matrix or distance measure exists for the objective comparison of two sets of protection factors. As such, important relationships could be overlooked, or worse, erroneous knowledge could be inferred from comparisons that separate the effects (*e.g.* comparing side chain identity in the absence of information about the thermodynamic stability at the same position).

One-dimensional software tools have been developed for the special case of hydrophobicity analysis, such as identification and alignment of the membrane spanning regions of non-globular proteins [26]–[28]. Although useful, these tools have historically incorporated family-specific scoring matrices [29] and empirical gap penalties. Such heuristics hinder the algorithms' transferability to different proteins or applicability to data types other than transmembrane protein hydrophobicity. In addition, the scoring functions for hydrophobicity analysis are often based on template-based matching or absolute similarity [30], and while this is effective at finding matches that are similar in both shape and magnitude, two sets of data that describe the same shape, but are offset by a scalar value, would be missed. For example, such a case can arise for experimentally measured local thermodynamic stabilities of proteins, where the relative stabilities of the same structural region of two homologs are observed to be strikingly similar, yet offset by a constant ΔΔG value [31]. Finally, some of these previous tools lack the capability for large database searches or do not include estimates of statistical significance, limiting their usefulness and effectiveness even for the appropriate input data.

To address these shortcomings, we have developed a tool to compare the internal consistency of one-dimensional profiles defined by arbitrary sequences of numerical data. To maximize the flexibility of the tool, we have deliberately chosen in the design to include two metrics that match both the relative shapes of the two profiles as well as the absolute similarity of the numerical values. Thus, the scoring system is designed to be independent of the input data type (as opposed to the tool's probability model which is very much dependent on the data type). Since this design emphasizes the closeness in shape of the two sets scanned over a horizontal range of positions, in contrast to the vertical position-by-position independent scoring of a standard amino acid substitution matrix, the algorithm is named *Horizontal Protein Comparison Tool* (*HePCaT*).

## Materials and Methods

### Detailed description of the *HePCaT* algorithm

**Figure 1:**
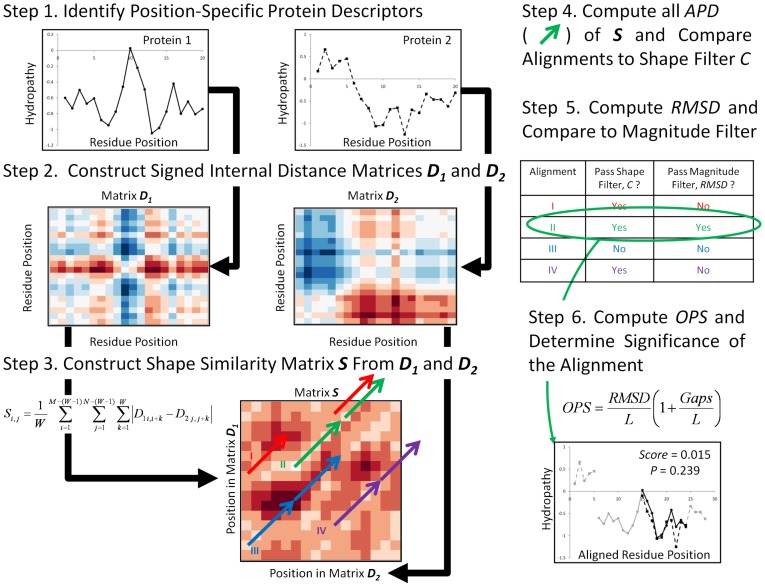
Overview of the Horizontal Protein Comparison Tool (*HePCaT*) algorithm. The hydropathy profiles of two hypothetical proteins, each of length *M* = *N* = 20 residues, are shown (Step 1). Intraprotein signed distances are computed within each protein according to Equation 1 in the main text (Step 2). Positive distances, *e.g*. measured from a residue with a local minimum value to a residue with a local maximum value, are indicated in red, negative distances in blue. The signed distance matrices are therefore square and symmetrically reflected across the diagonal. Distances for protein 1 and protein 2 correspond to matrices ***D_1_*** and ***D_2_***, respectively. The similarity matrix ***S*** that ultimately compares the two proteins is constructed from the average absolute distance differences of *W* = 5 residue blocks between ***D_1_*** and ***D_2_***, according to Equation 2 (Step 3). In ***S***, light colored squares indicate blocks of *W* = 5 residues starting at residue *i* in protein 1 and residue *j* in protein 2 with similarly shaped hydropathy, dark squares indicate dissimilar shapes. (***S*** *_i = 1,j = 1_* is the lower left corner in the figure.) As described in the text, ***S*** is exhaustively searched and all longest alignments with up to possibly *GapMax* gaps, whose squares (average path distance, *APD*) pass a user-defined average similarity cutoff *C*, are kept in a list (set of colored arrows). The alignment of this list with the closest absolute shape (lowest *RMSD*) is defined as the optimal match (Step 5). An Optimal Path Score (*OPS*), defined by Equation 4, is assigned to the alignment and its significance is computed with respect to the score distribution of random alignments of identical length (Step 6). Note that the example alignment, while a reasonable visual match, is only marginally significant with respect to random alignments of identical length, due to its short length of 10 residues.

The algorithm proceeds by creating internal signed distance matrices from each of two sets of input numerical data vectors *v* (Figure 1, Steps 1 and 2). The vector is composed of *M* elements given a protein of length *M* residues. In the following development, *v_i_* denotes an arbitrary numerical value at residue *i*. For a protein of *M* residues, each element of its distance matrix ***D*** is defined as
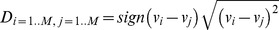
(1)The signed distance matrices, while not symmetric, are reflections across the diagonal (Figure 1, Step 2). Thus, both shape and magnitude information about each data set are encoded in these matrices. For example, the Protein 2 matrix ***D_2_*** (Figure 1, Step 2) clearly indicates the strong local maximum in the N-terminal half relative to the strong local minimum in the C-terminal half as prominent red or blue regions.

Equation 1 demonstrates a key conceptual difference from structure comparison algorithms that are usually based on distance or contact matrices restricted to only positive values [32], [33]. This difference reflects the nature of the information being compared. For structure comparison, the distance between two atoms is identical whether it is computed between the first and second atom or *vice versa*, while in the case of thermodynamic stability, for example, there may be a relative stabilization between the first and second atoms, which becomes a relative destabilization between second and first. The sign in Equation 1 thus represents this key conceptual difference: a “distance” in *HePCaT* has *both* sign and magnitude. (It is noted that Equation 1 may be extended to an arbitrary number of mathematical dimensions, but the present work only considers the one-dimensional case.)

A shape similarity matrix, ***S***, is then constructed from the two distance matrices (Figure 1, Step 3). To speed the calculation, a heuristic window size, *W*, is introduced. (In this work, *W* is always five residues, but we note that this is potentially an adjustable parameter and a completely exhaustive search may be performed with *W* = 1.) For each position *i = M−(W−1)* in Protein 1 and each position *j = N−(W−1)* in Protein 2, the relative shape similarity is computed between the two five-residue blocks originating at positions *i* and *j*:
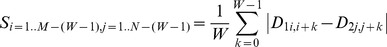
(2)
Equation 2 is simply the average absolute value of the difference of equivalenced internal distances between the two blocks. If the shape similarity is high this value will be small, if the shape similarity is very different this value will be large. Such dissimilarity can be readily viewed for the example proteins: the Figure 1 similarity matrix contains strong positive values (darkest red) where the large peak in the middle of the first protein coincides with the deep valley in the C-terminal region of the second (or *vice versa*).

In this implementation, the signed internal distances within each block of *W* = 5 residues are scaled such that the longest absolute value of the internal distance is one,
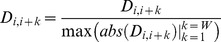
(3)Although this normalization can be disabled, we believe that emphasizing comparison of relative shape improves detection of relative trends in biological data, which can exhibit wide variations in scale. Practically, normalization also intuitively simplifies the choice of the user-defined alignment shape similarity cutoff, as described below.

The optimal alignment between Proteins 1 and 2 is found by exhaustive search of the shape similarity matrix (Figure 1, Steps 4 and 5). “Optimal” is defined as the largest unique set of blocks of size *W*, subject to at most *GapMax* skipped positions of the similarity matrix between blocks, which exhibits the smallest *RMSD* of all such sets passing a user-defined shape similarity cutoff, *C*. If *C* = 0, exact shape matches only are permitted in the alignment list. For this work, where Equation 3 applies, *C* was set to 0.40, meaning that an alignment whose average normalized distance between two five residue blocks was at most 40% different was counted as a matching shape. If Equation 3 were disabled, *C* would have to be adjusted empirically based on the dynamic ranges of data compared.

The algorithm starts at cell (1,1) of ***S*** (*i.e.* the lower left corner of the matrix in Figure 1, Step 3), corresponding to the average difference between the scaled intraprotein distances of residues 1–5 in Protein 1 and residues 1–5 in Protein 2. If ***S***_1,1_< = *C*, this match is kept and position ***S***_6,6_ is checked, until all cells of ***S*** are evaluated up to the position ***S*** *_M-W+1,N-W+1_* (*i.e.* the upper right corner of the matrix in Figure 1, Step 3). If at any point ***S***_i,j_>*C*, single cell gaps are inserted in one or both sequences up to a maximum of *GapMax* in an attempt to obtain the longest path through ***S*** subject to *C*. A list of the longest gapped paths is kept at this stage (Figure 1, Step 3, colored arrows). Therefore, all paths in this list are comprised of equivalenced positions in the two proteins such that, on average, the intraprotein distances seen at every position match to at least degree *C*; this average value is named Average Path Distance (*APD*, Figure 1, Step 4). *GapMax* was empirically set to 4 for this work. No penalty is applied to *APD* for insertion of a gap. Importantly, at this first stage only relative shape similarity is checked; any systematic offset between the two data sets is ignored because only the differences between intraprotein distances are evaluated.

After ***S*** has been exhaustively searched, the list of longest alignments passing the shape cutoff is filtered by *RMSD* of the aligned positions (Figure 1, Step 5). The smallest *RMSD* alignment is defined as the optimal (thus, the *RMSD* is effectively a magnitude filter). If multiple alignments of identical longest length happen to exhibit identical *RMSD*, only the first such one encountered is returned. In *HePCaT*, the *RMSD* calculation is executed after translation of both sets to data to their respective centers-of-mass, thus effects of a global offset between each data set are again minimized. Following Jia, *et al.* [34], we define an Optimal Path Score (*OPS*) for this optimal alignment according to the formula:
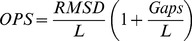
(4)In Equation 4, *L* is the alignment length and *Gaps* is the total number of cells skipped in ***S*** to obtain that alignment. Note that, as mentioned above, gaps are not explicitly penalized during alignment, but gaps will penalize the final score according to Equation 4, under the reasonable and common assumption that a gapless match is a “better” match than a gapped one. Alternatively, the *GapMax* parameter could be set to zero if desired so that all gaps are forbidden.

A probability model to estimate the significance of an *OPS* score *s* of an alignment of length *L* was derived from analysis of randomly generated alignments (Figure 1, Step 6). It is important to realize that a probability model is specific to the type of data aligned and must also be recalibrated for a specific combination of *W*, *C*, and *GapMax*. The probability model for Kyte-Doolittle hydropathy [35], averaged over a 15-residue window, is listed in Tables 1 and 2 and was built for the following *HePCaT* parameters: *W* = 5 residues, *GapMax* = 4 residues, *C* = 0.4 with the local scaling of Equation 3. (Other probability models have been constructed and tested by the authors, including models based on *eScape* predicted native state thermodynamic stability [19], and predicted translation efficiency index *tAI* [20], [21], and are available upon request.)

**Table 1:** Goodness of fit statistics between Scaled Inverse Chi Squared probability distribution function (Equation 5) and *OPS* score distributions of various length optimal *HePCaT* alignments of random amino acid sequences.

Hydropathy						
Kyte-Doolittle Hydropathy, averaged over 15 residues						
*W* = 5 residues						
*GapMax* = 4 residues						
*C* = 0.4						

*HePCaT* Alignment Length	*ν*	*ln σ^2^*	*χ^2^*	*d.o.f.*	*P-Value*	*N*
20	20.030	−4.092	19.4	20	0.37	433
25^a^						
30	20.444	−4.266	2.3	9	0.94	205
35	18.771	−4.305	9.8	15	0.71	322
40	22.152	−4.371	16.8	17	0.33	365
45						
50	23.895	−4.507	10.2	14	0.60	309
55	31.086	−4.556	23.7	17	0.07	368
60	27.883	−4.634	17.9	19	0.39	414
65	31.871	−4.675	9.1	17	0.87	379
70	34.017	−4.751	11.3	15	0.58	339
75	37.144	−4.752	16.0	19	0.52	405
80	40.667	−4.860	15.5	19	0.56	419
85	39.468	−4.851	19.2	17	0.21	374
90	40.866	−4.903	15.1	16	0.37	343
95	50.460	−4.935	19.0	18	0.27	386
100	58.710	−4.974	16.3	16	0.29	352
105	48.502	−5.033	15.0	15	0.31	329
110	50.481	−5.038	5.4	11	0.80	254
115	60.850	−5.074	6.9	14	0.86	315
120	52.309	−5.114	8.6	12	0.57	267
125	56.929	−5.160	7.4	13	0.76	295
130	73.921	−5.170	11.6	12	0.31	279
135	66.086	−5.231	3.7	13	0.98	282
140	91.441	−5.262	8.4	11	0.50	251
145	75.360	−5.265	4.6	12	0.92	276
150	74.003	−5.289	5.2	13	0.92	296
155						
160	82.535	−5.341	8.7	14	0.73	308
165	74.069	−5.378	7.9	15	0.85	331
170	87.990	−5.403	12.0	14	0.45	319
175	78.128	−5.437	19.1	17	0.21	362
180	84.227	−5.449	22.2	17	0.10	360
185	92.662	−5.472	9.8	15	0.71	332
190	85.812	−5.493	12.0	16	0.61	343
195	86.967	−5.531	12.7	16	0.55	344
200	108.592	−5.540	12.5	14	0.41	319
205	104.753	−5.565	13.1	15	0.44	332
210	109.308	−5.603	9.8	14	0.64	317
215						
220	103.593	−5.631	11.9	12	0.29	262
225	106.655	−5.651	9.2	12	0.51	260
230	108.842	−5.658	5.1	9	0.65	213
235	106.144	−5.687	9.1	9	0.25	203
240	147.619	−5.705	6.2	9	0.52	201
245	111.964	−5.717	4.7	7	0.45	173

a Blank rows for certain alignment lengths indicate that the null hypothesis (*i.e.* that the distribution of *OPS* scores for randomly generated sequences was drawn from an underlying inverse chi square distribution) was rejected at the *p*<0.05 level.

**Table 2:** Parameters used in Equations 6 and 7 to estimate length-dependent random protein data probability distributions based on the Inverse Chi-Squared Distribution.

Data Type	*m*	*a*	*b*	*c*
Hydropathy	0.497609	0.160379	−1.04167	38.9045

### Construction of probability model

**Figure 2:**
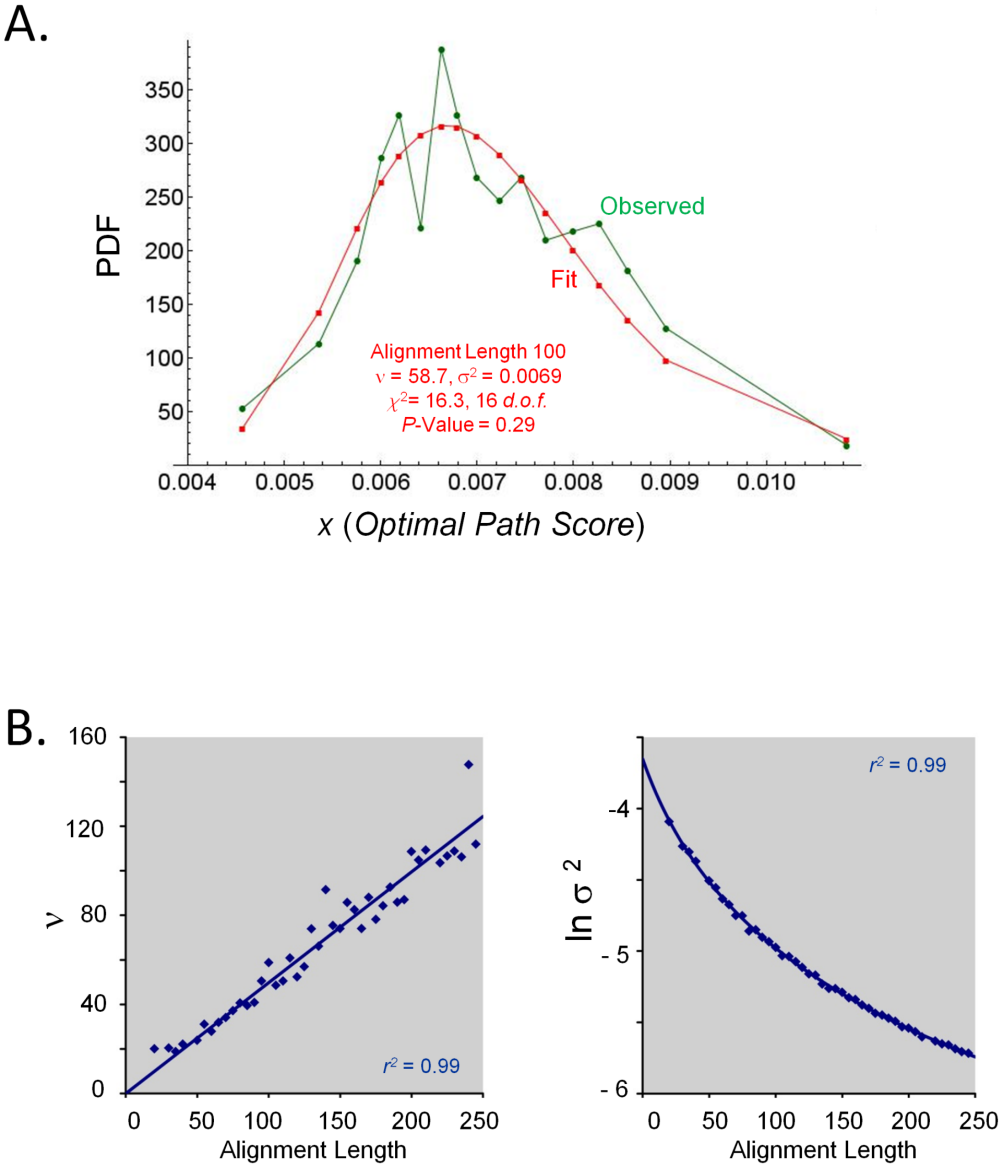
Empirically determined probability model for protein hydropathy. **A.** **Inverse Chi-Squared model for the distribution of observed scores.** Distributions of Equation 4 scores for *HePCaT* alignments of length *L* = 100 obtained from parameters *W* = 5 residues, *GapMax* = 4 residues, *C* = 0.4. Pairs of random sequences were generated, their Kyte-Doolittle amino acid hydropathies averaged over a 15-residue window, and subjected to optimal alignment using *HePCaT*, as described in the text. Binned data in each case was reasonably fit to the Inverse Chi-Squared probability distribution function (PDF, Equation 5), as described in Methods and tabulated in Table 1. **B.** **Analytical parameters to estimate statistical significance.** Parameters *ν* and *σ^2^* for the PDF were observed to vary smoothly as a function of *HePCaT* alignment length, allowing the parameters, and thus alignment significance, to be analytically estimated for arbitrary alignment length using Equations 6 and 7 and parameters in Table 2. Discrete best-fit parameters for *ν* and *σ^2^* are given in Table 1. Equations for displayed best-fit curves are as follows: y = 0.497609x (Hydropathy, *ν*), y = 0.160379–1.04167 ln(x+38.9045) (Hydropathy, *σ^2^*).

Significance of the Equation 4 score of optimal *HePCaT* alignments was estimated with respect to the scores of optimal alignments of identical length between proteins of random amino acid sequence. Two random proteins of equal lengths between 10 and 500 residues were generated according to background amino acid frequencies as given by Robinson & Robinson. [36] Sets of at least 20,000 such pairs for each length were optimally aligned using *HePCaT*, and the distributions of Equation 4 scores for a given optimal alignment length from the entire pool were tabulated (Figure 2A). It was observed that these skewed unimodal distributions exhibited a strong dependence on alignment length (Figure 2B). Out of several possible two-variable formulae, it was empirically determined that these score distributions were statistically best fit by Scaled Inverse Chi-Squared probability density functions (Figure 2, Tables 1 and 2) [37],
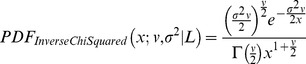
(5)In Equation 5, *L* is optimal alignment length, and Γ(x) is the Gamma function. [38] Parameters ν and σ^2^ were estimated by minimum chi-squared fits to the binned score data at each observed alignment length (Figure 2A). Binning and parameter estimation were performed using custom *Mathematica* 8.0 scripts, such that each variable-width bin contained at least 20 points, additional details are provided in Table 1.

*Ad-hoc* analytical expressions were fitted to the collected best-fit parameters of Equation 5 as a function of optimal alignment length *L* (Figure 2B):
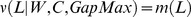
(6)

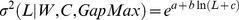
(7)Determination of coefficients *a*, *b*, *c*, and *m* only employed reasonably well-fit Equation 5 values whose null hypotheses (*i.e.* that the simulated data were drawn from Inverse Chi Square Distributions) could not be rejected at *p*<0.05. Equations 6 and 7 coefficients for protein hydropathy are given in Table 2, all resulted from excellent fits of *R^2^* = 0.99 or better using *gnumeric* spreadsheet software (Figure 2B).

Therefore, given an observed optimal *HePCaT* alignment of length *L* with Equation 4 score *s*, the probability *p* of observing that alignment of protein hydropathy by chance could be estimated from the corresponding Scaled Inverse Chi-Squared cumulative distribution function as:
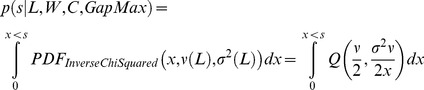
(8)In Equation 8, *Q(a,x)* is the complement of the regularized Gamma function [38]; *ν* and *σ^2^* were estimated from Equations 6 and 7, using coefficients of Table 2.

### Clustering of membrane protein structures based on hydropathy

All 1604 amino acid sequences corresponding to every membrane protein structure in SCOP 1.73 (class *f* ) [39] were obtained from the ASTRAL domain database [40] and clustered at 70% sequence identity by the cd-hit server [41], resulting in 214 representative sequences. The Kyte-Doolittle hydropathy values [35] for each sequence were averaged over a window size of 15 residues, with the average being assigned to the middle position of the window. These 214 hydropathy profiles were then compared using *HePCaT* in an all-*vs*-all manner, with the probability value for each optimal match computed using the model coefficients listed in Table 2. For each protein, a vector of length 214 containing the probability values against all other proteins was constructed. These 214 vectors were then clustered by Manhattan Distance and Ward's minimum variance criterion as implemented in the Hierarchical Clustering Package of *Mathematica 8.0* (Wolfram Research) to create a dendrogram. A similar tree was computed from *FASTA* [42] E-values of all pairwise sequence comparisons. Significance of each grouping was estimated using the bootstrap “Gap Test” option of the software.

### Hydropathy database search of the human proteome using adenosine receptor A2a as query

The human proteome was obtained from translation of the DNA sequences contained in the NCBI CDDS [43] build 36.3 (April 30, 2008). Each amino acid in every protein was assigned a value according to the Kyte-Doolittle hydropathy scale.[35] The values for each protein were averaged using a 15 residue sliding window; averaged values for the first and last seven residues in each protein were subsequently ignored. The averaged values for the G-protein coupled receptor (GPCR) human adenosine receptor A2a (CCDS 13826.1, gi|5921992) were used as query against the human proteome, *i.e.* the averaged hydropathy values of each protein in the proteome were optimally pairwise aligned to A2a using *HePCaT* with the following parameters: *W* = 5 residues, *C* = 0.4, *GapMax* = 4 residues. *P*-values for each alignment were computed using the probability model specific to these data as described above. GPCRs were checked and annotated in our local copy of the human proteome by *FASTA*-aligning [42] amino acid sequences of the proteome with amino acid sequences of known GPCRs obtained from the *GPCRDB* [44]. Modeling was performed with a local installation of I-TASSER software [45] using default parameters. Structural similarity between the first I-TASSER model and known proteins was assessed using the DALI server [46].

### Discovery of similarity between *ORFan* protein *TC0624* and colicin pore-forming domain

A dataset of 8812 ORFan protein sequences was obtained from Yomtovian, *et al.* [47] As described above, *HePCaT* was used to optimally align the Kyte-Doolittle averaged hydropathy profiles of each ORFan protein with the profile of each member of the non-redundant set of 214 membrane proteins of known structure described above Secondary structure prediction was performed using the PSIPRED server [48] [49] and Hidden Markov Model sequence profile comparison was performed using the HHpred server [50], both with default parameters. Modeling was performed with a local installation of I-TASSER software [45] using default parameters. Structural similarity between the first I-TASSER model and known proteins was assessed using the DALI server [46].

## Results

The biological utility of *HePCaT* was assessed by exploring three different questions relating to protein hydropathy: sequence clustering of known membrane protein structures, similarity search against a database, and structure prediction of an ORFan protein. Results described below provided biological insight and testable hypotheses from these common bioinformatics tasks. However, it is emphasized that the results are not intended to demonstrate improvement of *HePCaT* over current state-of-the-art methods for sequence and structure comparison, rather, the results do illuminate strengths and weaknesses of the algorithm's current implementation.

### Clustering of known membrane protein structures based on common hydropathy patterns

Unlike most globular proteins, most membrane protein structures can be classified, independent of evolutionary relationships, into two main groups, “all-alpha” and “all-beta”, based on structural characteristics alone [51], [52]. One dominant characteristic is the requirement for stability within the nonpolar interior of the membrane, and this is reflected in recurring patterns of defined length hydrophobic segments, imposed by the physical constraints of alpha-helical or beta-strand secondary structure elements. Such patterns can be used for the effective prediction of transmembrane spanning segments and fold topology of the inserted protein [53]–[55].

**Figure 3:**
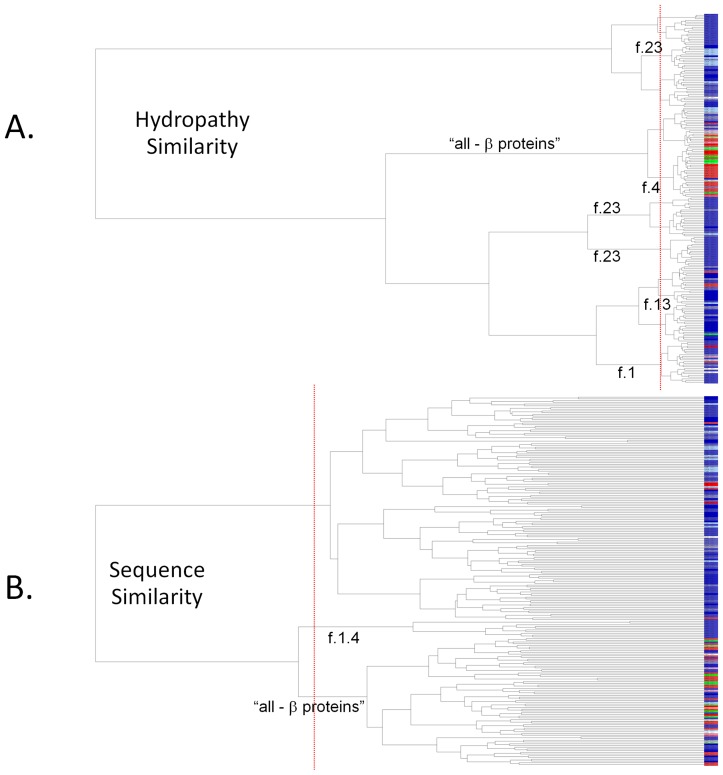
Clustering of known membrane protein structures by hydropathy similarity. Dendrogram leaves are members of a set of 214 representative membrane protein structures taken from SCOP 1.73, as described in the text. Blue colors denote proteins of all (or mostly) alpha helical secondary structure, red colors denote proteins of all (or mostly) beta strand secondary structure, and green colors indicate proteins of mixed structure. Identical shades of color denote identical SCOP fold. Particular sub-branches that significantly cluster according to known evolutionary or structural relationships are labeled by SCOP fold. Vertical dashed red lines indicate statistical significance of the clustering. **A.** **Dendrogram based on hydropathy similarity.** Branch lengths are inversely proportional to the *HePCaT* significance of the pairwise similarity between hydropathy patterns (*i.e.* shorter branch lengths indicate higher similarity). **B.** **Dendrogram based on sequence similarity.** Branch lengths are inversely proportional to FASTA E-value of pairwise sequence similarity. For these diverse proteins, both sequence and hydropathy similarity differentiate beta proteins from alpha proteins. However, the *HePCaT* beta dendrogram cluster is evidently more homogenous than the *FASTA* beta cluster, and more individual protein folds are segregated based on hydropathy similarity than by sequence similarity. Both observations suggest that meaningful information about protein structure and evolution can be objectively detected by the *HePCaT* algorithm.

Analysis and clustering of a set of diverse membrane protein structures, based on similarities in the proteins' average hydropathy patterns using *HePCaT*, reflects this major level of structural organization (Figure 3A). In this dendrogram, the “all-beta” proteins clearly segregate into distinct and statistically significant sub-branches of the tree. Finer levels of overall fold similarity, including the G-protein coupled receptors (f.13), toxins' membrane translocation domains (f.1), and the transmembrane beta barrels (f.4), can also largely be resolved only on the basis of hydropathy similarity (labeled sub-branches in Figure 3A). Interestingly, proteins belonging to f.13, annotated as “single transmembrane helix” and thus “not a true SCOP fold” [56], are spread among several dispersed sub-branches, consistent with this provisional expert curation.

In contrast, clustering of the identical proteins based on pairwise amino acid sequence similarity alone appears less resolved at levels higher than pairs of highly similar sequences (Figure 3B). In particular, the “all-beta” proteins, while also resolved to a particular statistically significant sub-branch, are not cleanly segregated from other “all-alpha” proteins. Few fold families are clustered at statistical significance, probably due to the overall low level of sequence similarity in this diverse set (approximately 30% identity over 40 residues on average). Clearly, patterns of hydropathy, reflecting the well-known idea that protein structure similarity is more conserved than sequence similarity [57], [58], can be objectively recovered using pairwise *HePCaT* alignments in conjunction with the appropriate probability model described above.

### Database search using human adenosine receptor A2a as query

**Figure 4:**
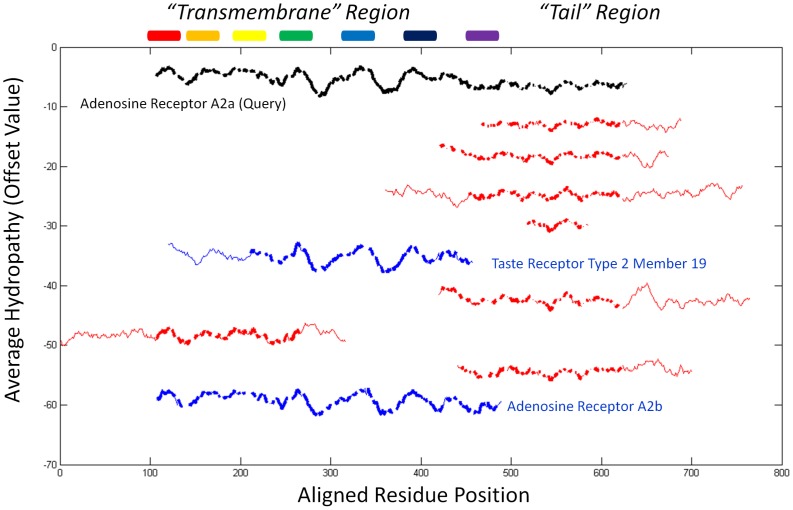
Most significant similarities in the human proteome to the Kyte-Doolittle hydropathy profile of adenosine receptor A2a. Pairwise *HePCaT* alignments are shown for A2a (black, gi|5921992) and the top nine most significant nonredundant hits in the human proteome. Blue color indicates known seven transmembrane spanning region proteins as annotated by the *GPCRDB* database, red mostly indicates hits to the tail region of A2a. The hits are shown from top to bottom in order of most to least significant: hematological and neurological expressed protein-like 1 (gi|21700763, *p* = 4.0×10^−6^), ephrin-A4 isoform a precursor (gi|4885197, *p* = 7.6×10^−5^), NSFL1 cofactor p47 isoform a (gi|20149635, *p* = 9.1×10^−5^), metallothionein-1E (gi|83367075, *p* = 9.7×10^−5^), taste receptor type 2 member 19 (gi|28882035, *p* = 4.1×10^−4^), B- and T-lymphocyte attenuator isoform 1 precursor (gi|145580621, *p* = 5.4×10^−4^), WD-repeat domain-containing protein 83 (gi|153791298, *p* = 6.5×10^−4^), dual specificity protein phosphatase 26 (gi|13128968, *p* = 7.7×10^−4^), adenosine receptor A2b (gi|4501951, *p* = 8.3×10^−4^). Thick lines indicate residue positions included in the optimal *HePCaT* alignment to A2a, and thin lines indicate unaligned positions. Rainbow colored cylinders from N- to C-terminus indicate the approximate sequence locations of the seven experimentally determined transmembrane spanning helices of A2a.

Given the ability of *HePCaT* to match expected hydropathy patterns, an exploratory search was initiated to discover unknown matches. The hydropathy profile of the human adenosine A2a 7Tm G-protein coupled receptor (GPCR) was used to search the human proteome for close unreported matches. As expected, hundreds of known 7Tm GPCRs were significantly matched by *HePCaT* (*p*<0.01, data not shown). The most significant ten matches are displayed in Figure 4. These hits fell into two categories: those that matched the transmembrane region [59] of A2a (Figure 4, blue) and those that mostly matched the tail region (Figure 4, red).

**Figure 5:**
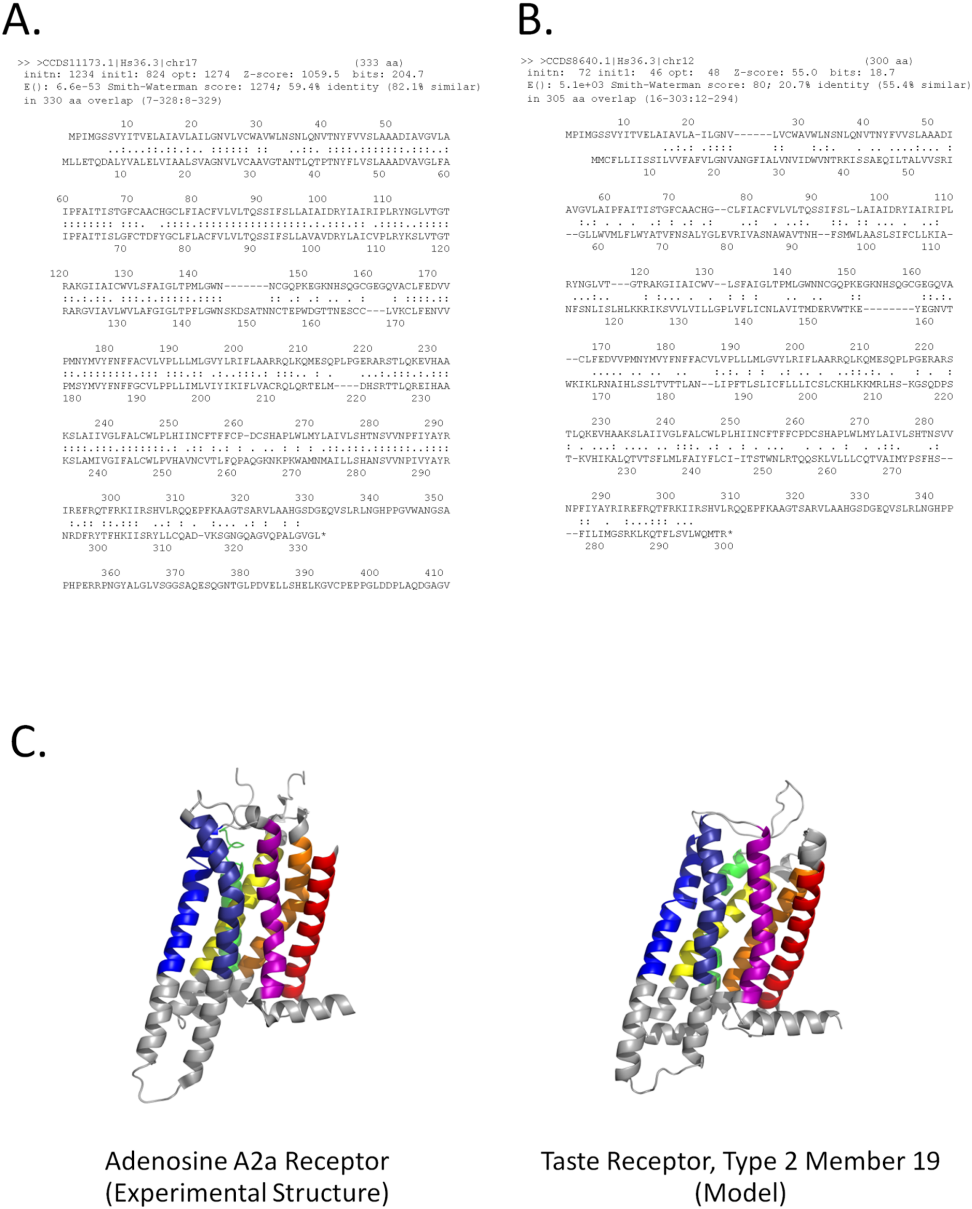
Pairwise sequence alignment does not detect significant similarity between human A2a and Taste Receptor Type 2, Member 19, yet a similar structure can be modeled based on the *HePCaT* match. **A.** ***FASTA* pairwise sequence alignment between human adenosine receptor A2a and its known homolog human adenosine receptor A2b.** Alignment was extracted from a sequence search of the human proteome. Sequence similarity is 59% over 330 amino acids, with a highly significant E-value of 6.6e-53. Note that the hydropathy similarity between these two proteins is also significant, as given in Figure 4. **B.** ***FASTA*** **pairwise sequence alignment between human A2a and human taste receptor type 2, member 19.** Sequence similarity is 21% over 305 amino acids. Although extensive, the similarity is not significant, with an E-value of 5.1e+3, in contrast to the significant hydropathy similarity displayed in Figure 4. This result suggests that hydropathy similarity, as assessed by *HePCaT*, may be able to detect remote relationships in the absence of sequence similarity. **C.** **Model of Taste Receptor Type 2, Member 19 is similar to the experimental structure of A2a.** Experimental structure of A2a (left panel) is based on PDB identifier 3rey. I-TASSER [45] model of Taste Receptor Type 2, Member 19 (right panel) achieved an I-TASSER C-score of 0.67 and a DALI Z-Score [46] of 24.9 against the 3rey structure, indicating a confident model that is significantly similar to A2a. Rainbow colored helices follow the colors of Figure 4, indicating the seven structurally aligned transmembrane spanning helices. The RMSD of the 269 DALI-aligned residues is 3.1 Å between modeled and experimental structures.

The longest match to the transmembrane region was the A2b isoform, which is also 59% sequence identical to A2a (Figure 5A). Unexpectedly, a Type 2 taste receptor also exhibited a significant match to this region (Figure 4). As this taste receptor has insignificant pairwise sequence identity to A2a (Figure 5B) and its structure has not been experimentally determined [60], this observed similarity was consistent with an independently produced model of the taste receptor, constructed using no *HePCaT* information (Figure 5C). Additionally, the original *HePCaT* match was demonstrated to be a useful template for a homology model [61] based on the A2a structure (data not shown). The validity of the hydropathy similarity between A2a and the taste receptor was further demonstrated to be robust with respect to the particular hydrophobicity scale used (Text S1; Figures S1 and S2 in Text S1).

We attempted to rationalize the best matches to the A2a tail region in terms of sequence, structure, or function. However, in contrast to the transmembrane region matches, biological explanations for these remain unknown. The shortest hit to the tail region was possibly a statistical artifact: this metallothionein is naturally short and contains a high frequency of cysteine residues; such low-complexity sequences are normally filtered out of amino acid sequence searches [62], which was not done in the present study. Some of the proteins in this group are medically important, such as the hematological and neurological expressed-1 like protein, ephrin A4 isoforms, and the B and T-lymphocyte attenuator precursor. Structural information, where available about the matches, could not be confidently transferred to the putatively disordered tail region of A2a, which is thought to be involved in ligand specificity of the GPCR [63]. These tail matches may also result from the local scaling (Equation 3), which could potentially be disabled, illustrating the sensitivity *vs.* specificity tradeoffs inherent to relative shape matching.

### Predicted remote similarity between the pore forming domain of bacterial colicin and Chlamydia *TC0624* protein

**Figure 6:**
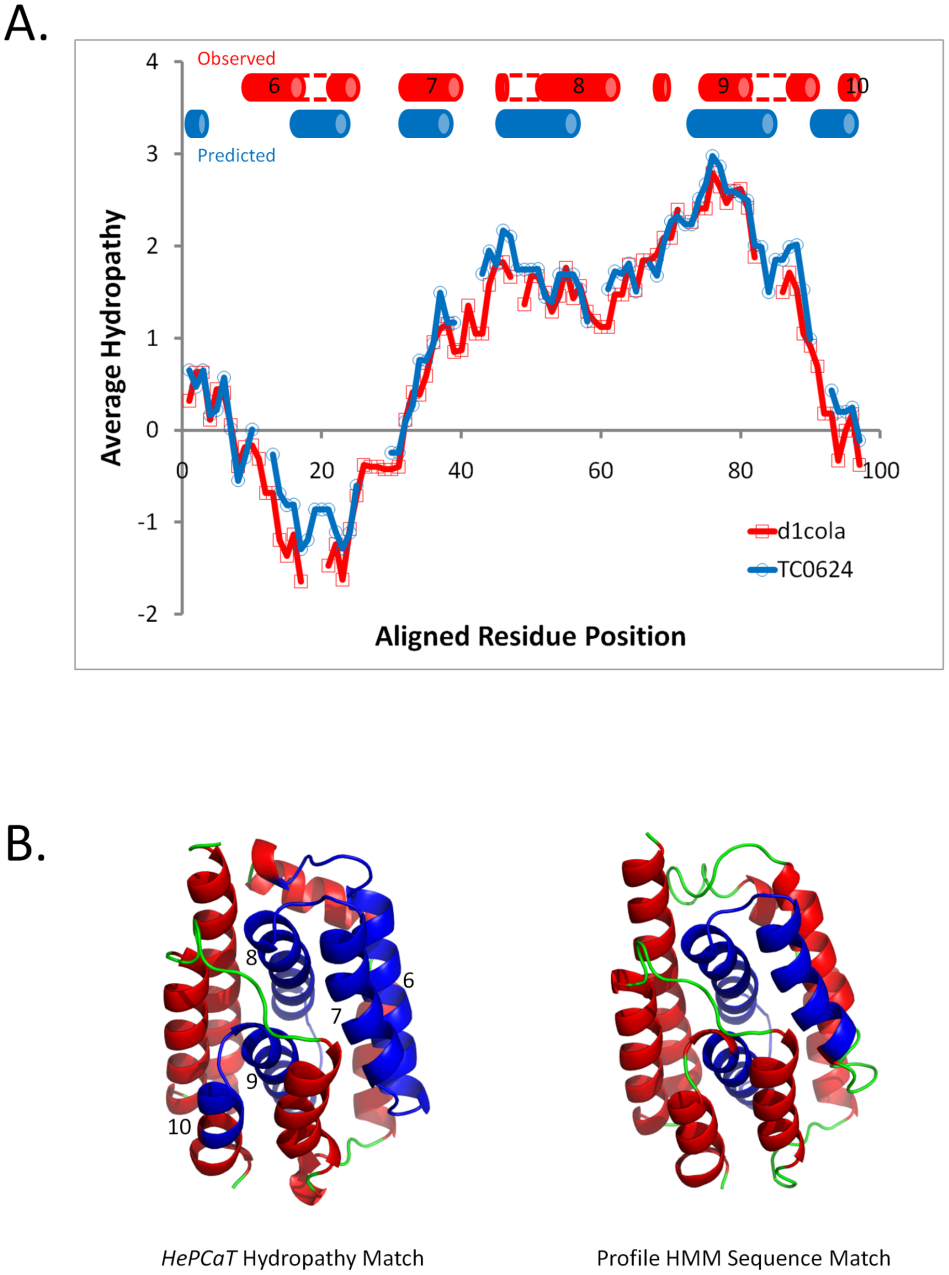
Observed hydropathy and predicted structure similarity between ORFan *C. muridarum TC0624* and bacterial colicin pore-forming domain. **A.** **Significant similarity between hydropathy of *TC0624* and *E. coli* colicin A (SCOP domain d1cola_).** The likelihood of obtaining this match by chance is *p* = 1.5×10^−5^. The blue cylinders indicate PSIPRED confidently predicted helical secondary structure of TC0624, the red cylinders indicate the actual helical secondary structure of d1cola_ domain as assessed by DSSP [69]. Numbers indicate the functionally important helical elements, as annotated by Cramer, et al. [65] Reasonable correspondence between the type and locations of secondary structure elements is observed. Gapped regions of colicin helices are connected with dotted lines to guide the eye. **B.** **Tertiary structure location of the hydrophobic similarity (left) and the sequence similarity (right) matches between** ***TC0624*** **and colicin.** In both molecular cartoons, helices are colored red, strands yellow, and loops green. Locations of a match between *TC0624* and colicin are colored blue. The left figure is based on d1cola_, colored according to the *HePCaT* alignment in Figure 6A, and the right figure is based on the homolog d1rh1a2 SCOP domain observed in the marginally significant *HHPred* [50] hidden Markov model sequence match. Both matches independently link the sequence and hydrophobicity of the ORFan to the functionally important structural core region of colicin. The extensive structure, sequence, and chemical similarities suggest the medically important hypothesis that *TC0624* could also be a pore-forming protein facilitating chlamydia survival.

A third example of the utility of *HePCaT* concerns the possible discovery of remote similarity with medical importance. The *C. muridarum* protein *TC0624*, classified as an “ORFan” due to the absence of significant sequence similarity between any other known proteins [47], nonetheless exhibited a significant *HePCaT* hydropathy match to the pore forming domain of *E. coli* colicin A (Figure 6A). This match spanned the entire chain length of the ORFan protein and the experimentally-determined minimal length region of functional importance of the pore-forming domain [64]. The validity of the hydropathy similarity between colicin and TC0624 was further demonstrated to be robust with respect to the particular hydrophobicity scale used (Text S1; Figures S1 and S2 in Text S1).

Secondary structure prediction was consistent with the proposed tertiary structural similarity (Figure 6A), and sensitive sequence profile search using hidden Markov models revealed marginal (maximum *HHPred* P-Value 30% [50]), but repeated, similarity to the sequence of colicin implicated in the hydropathy match (Figure 6B). Thus, a total of four lines of evidence (hydropathy, secondary structure prediction, sensitive sequence similarity, and the regional correspondence between the sequence and structure matches) all converged on similarity between *TC0624* and the pore forming domain of colicin. Modeling [45] of *TC0624* also resulted in a low-confidence fold prediction consistent with colicin (data not shown). However, these conclusions would have not been possible without the original statistical significance of the *HePCaT* hydropathy match.

Importantly, the hydrophobic region of colicin implicated in this match has long been thought to be functionally crucial for colicin's lethal ability to travel from a hydrophilic extracellular environment, insert into the hydrophobic membrane interior, and form toxic pores in its host [65]. *TC0624* has independently been placed [66] in a class unique to *Chlamydiae* that is observed by experiment to also similarly partition into the membrane interior of the chlamydial inclusion [67]. These so-called “*Inc*” proteins, difficult or impossible to predict using existing computational tools [66], are nonetheless important for chlamydial survival and maturation within its human or animal hosts. It appears that the extreme hydrophobicity exhibited by the *Inc* proteins [67] facilitates their computational prediction using *HePCaT*.

Taken together, the results suggest a novel functional hypothesis for these medically important proteins: the *Incs* may form membrane-spanning pores that obtain nutrition from the host cytoplasm. This example also suggests that this particular ORFan may actually belong to a known protein family. Experiments are currently in progress to test these hypotheses.

## Discussion

Most protein and nucleic acid data contained within the avalanche of next-generation genome sequencing can be expressed as sequentially numeric “peaks” and “valleys”. These data include, but are not limited to, gene expression, ribosomal profiling, *ChIPSeq*, *RNASeq*, mRNA translation efficiency, thermodynamic stability of protein or mRNA, and physico-chemical properties such as hydropathy. A gap exists among software algorithms for analysis of such data, and the *HePCaT* algorithm described in this work is designed to help fill this gap. To facilitate such analysis and discovery, a webtool that allows execution of the algorithm, visualization of the result, and access to the raw and analyzed data is freely available at http://best.bio.jhu.edu/HePCaT. (A detailed manuscript describing the use and capabilities of this web portal is in preparation.) Due to patent and license restrictions, information about access to source code is available through The Johns Hopkins University Office of Technology Transfer from the corresponding author.

There are at least three distinguishing features of the *HePCaT* algorithm. First, the input is completely arbitrary: if the data can be expressed in numeric form regardless of its source, patterns can potentially be detected. Second, its scoring system is sensitive to both shape and magnitude similarity, allowing some degree of pairwise alignment flexibility. Third, the *W* parameter emphasizes a horizontal matching of patterns, as contrasted with the vertical matching that commonly occurs with amino acid substitution matrices or profile PSSMs.

In our view, vertical evolutionary conservation of amino acids has been thoroughly explored using tools such as *BLAST* [4], [5] and *FASTA* [42], while horizontal conservation of other protein properties has not. Thus, non-local properties of proteins, depending on correlations across residue positions, such as thermodynamic stability, can now be potentially explored with *HePCaT*. The case studies presented in Figures 5 and 6 suggest that substantial horizontal similarity can be detected in one pass through a database, minimizing the need for longer iterative searches when the vertical similarity may be weak or statistically impossible to detect.

Importantly these anecdotal examples are not intended to demonstrate the superiority of the *HePCaT* algorithm, or the information contained in horizontal conservation, over current state-of-the-art methods for remote homology detection that are based on vertical conservation. To the contrary, *HePCaT* is intended as a complementary tool that would be most usefully applied to cases where vertical conservation is weak or absent.

Furthermore, although the tool formally returns a pairwise positional alignment, it is not clear if such an alignment, could or should be quantitatively compared to existing amino acid sequence alignment tools. The *HePCaT* input is subject to possible averaging over one window size (*e.g.* the hydropathy is averaged over 15 positions) and the output is matched using quantized blocks of a second multi-residue window size (*e.g.* 5 positions). Future work is necessary to determine whether *HePCaT* can substantially improve upon the accuracy of the best current pairwise alignment methods.

Rigorous evaluation of the statistical significance of a result is an essential piece of scientific data that is often neglected in bioinformatics tools. The significances returned by *HePCaT* allow prioritization of matches and aid expert interpretation. As with other tools, the *HePCaT* statistical significances require calibration specific to the input data and algorithm parameters. Although recalibration for random simulation data not covered by Table 2 parameters is straightforward and has been achieved for other types of numerical data, an alternative estimate of statistical significance is available. Specifically, the non-parametric statistics of the *MIC* score reported by Reshef, *et al.* [68] could potentially be used to evaluate a match returned by *HePCaT*. In this way, the significances of arbitrary pattern associations reported by Reshef, *et al.* could be greatly leveraged by using *HePCaT* as a “front-end” for other types of numerical data. Although this idea has not yet been thoroughly studied, we believe that the applicability of the *MIC* statistics would be maximized with *HePCaT* parameters of *GapMax* = 0 and *W* = 1.

## Supporting Information

Text S1 **Significant** ***HePCaT*** **matches are robust to different hydrophobicity scales.**
(DOC)
