# Supplementary material for: A Horizontal Alignment Tool for Numerical Trend Discovery in Sequence Data: Application to Protein Hydropathy
Source: PLoS Comput Biol. 2013 Oct 10;9(10):e1003247. doi: 10.1371/journal.pcbi.1003247 (PMC3794901; doi:10.1371/journal.pcbi.1003247)
Supplement: Text S1 — Significant HePCaT matches are robust to different hydrophobicity scales. (DOC) [file pcbi.1003247.s001.doc]

**Supporting Information for Hadzipasic, *et al.* “A Horizontal Alignment Tool for Numerical Trend Discovery in Sequence Data”.**

**Text S1: Significant *HePCaT* matches are robust to different hydrophobicity scales.**

**Introduction**

The purpose of this Supporting Material is to document the robustness of two significant *HePCaT* matches discovered using the Kyte-Doolittle Hydropathy scale [1] with respect to other common, yet diverse, hydrophobicity scales. The original matches reported in the main text were between human adenosine receptor A2a (gi|5921992) and human taste receptor type 2, member 19 (gi|28882035), and between the pore-forming domain of *E. coli* colicin A (SCOP [2] domain d1cola_) and ORFan protein [3] TC0624 from *C. muridarum* (gi|7190664). These matches are displayed in Figures 4 and 6 of the main text, respectively.

**Materials and Methods**

These two pairwise comparisons were repeated using hydrophobicity profiles made from four scales other than Kyte-Doolittle. These scales were subjectively selected based on visual inspection of the comprehensive clustering of most known hydrophobicity scales, shown in Figure 4 of AAindex (http://www.genome.jp/aaindex). [4] The selected scales, unlike the Kyte-Doolittle scale, are centrally located in said clustering, but are nonetheless dispersed from each other and from the Kyte-Doolittle scale. These criteria were used in an attempt to bias the comparisons away from those reported in the main text, thereby testing whether the matches were strongly dependent on one particular scale.

As described, these profiles were all constructed using a window averaging of 15 residues. The scales were derived from either different experimental methods or computational analysis of protein structures as taken from the literature. For visual comparison to the Kyte-Doolittle scale, the signs of values were uniformly negated from the published scale if necessary. The values used in these calculations are given in Table S1.

**Table S1. Four diverse hydrophobicity scales.**

| Amino Acid | Radzicka-Wolfenden [5] ^a^ | Nozaki-Tanford [6,7] ^a^ | Fauchere-Pliska [8] ^a^ | Rose, *et al.*[9] |
| --- | --- | --- | --- | --- |
| W | -1.39 | -3.4 | -2.25 | 0.85 |
| F | -2.04 | -2.5 | -1.79 | 0.88 |
| Y | 1.08 | -2.3 | -0.96 | 0.76 |
| M | -1.41 | -1.3 | -1.23 | 0.85 |
| L | -3.98 | -1.8 | -1.70 | 0.85 |
| I | -3.98 | -1.8 | -1.80 | 0.88 |
| V | -3.10 | -1.5 | -1.22 | 0.86 |
| A | -0.87 | -0.5 | -0.31 | 0.74 |
| C | -0.34 | -1.0 | -1.54 | 0.91 |
| G | 0.0 | 0.0 | 0.0 | 0.72 |
| P | 0.0 | -1.4 | -0.72 | 0.64 |
| T | 3.51 | -0.4 | -0.26 | 0.70 |
| S | 4.34 | 0.3 | 0.04 | 0.66 |
| N | 7.58 | 0.2 | 0.60 | 0.63 |
| Q | 6.48 | 0.2 | 0.22 | 0.62 |
| D | 9.66 | 2.5 | 0.77 | 0.62 |
| E | 7.75 | 2.5 | 0.64 | 0.62 |
| H | 5.60 | -0.5 | -0.13 | 0.78 |
| R | 15.86 | 3.0 | 1.01 | 0.64 |
| K | 6.49 | 3.0 | 0.99 | 0.52 |

a. The numerical values for these three scales are taken from Creighton. [10]

**Results and Discussion**

The effects of different hydrophobicity scales were first assessed by comparing how they affected the profiles of each protein individually. This assessment was quantified using Pearson correlations [11] of the average hydrophobicity values at each position in the protein, computed over all possible pairs of scales. The results are displayed in Table S2, and suggest that the Rose, *et al.* scale is most highly correlated with Kyte-Doolittle, while the Nozaki-Tanford scale is among the least correlated. However, the lowest correlation coefficient in Table S2 is 0.75, indicating that all scales contain similar information to Kyte-Doolittle when the averaging over 15 residues is performed. Examples of this worst correlation, as well as the best, are given in Figure S1 to give a visual sense of the differences in information content.

**Table S2. Pearson correlation coefficients (R^2^) between Kyte-Doolittle hydropathy profile and profiles computed from other hydrophobicity scales for identical proteins**.

| Protein Name | Radzicka-Wolfenden | Nozaki-Tanford | Fauchere-Pliska | Rose, *et al.* |
| --- | --- | --- | --- | --- |
| Human A2a Receptor | 0.88 | 0.75 | 0.85 | 0.90 |
| Human Taste Receptor Type 2 Member 19 | 0.95 | 0.79 | 0.89 | 0.92 |
| *E. coli* Colicin | 0.92 | 0.77 | 0.86 | 0.91 |
| ORFan TC0624 | 0.93 | 0.89 | 0.97 | 0.97 |

**Figure S1. Examples of best and worst correlations between different hydrophobicity scales suggest little loss of hydropathy profile information.**


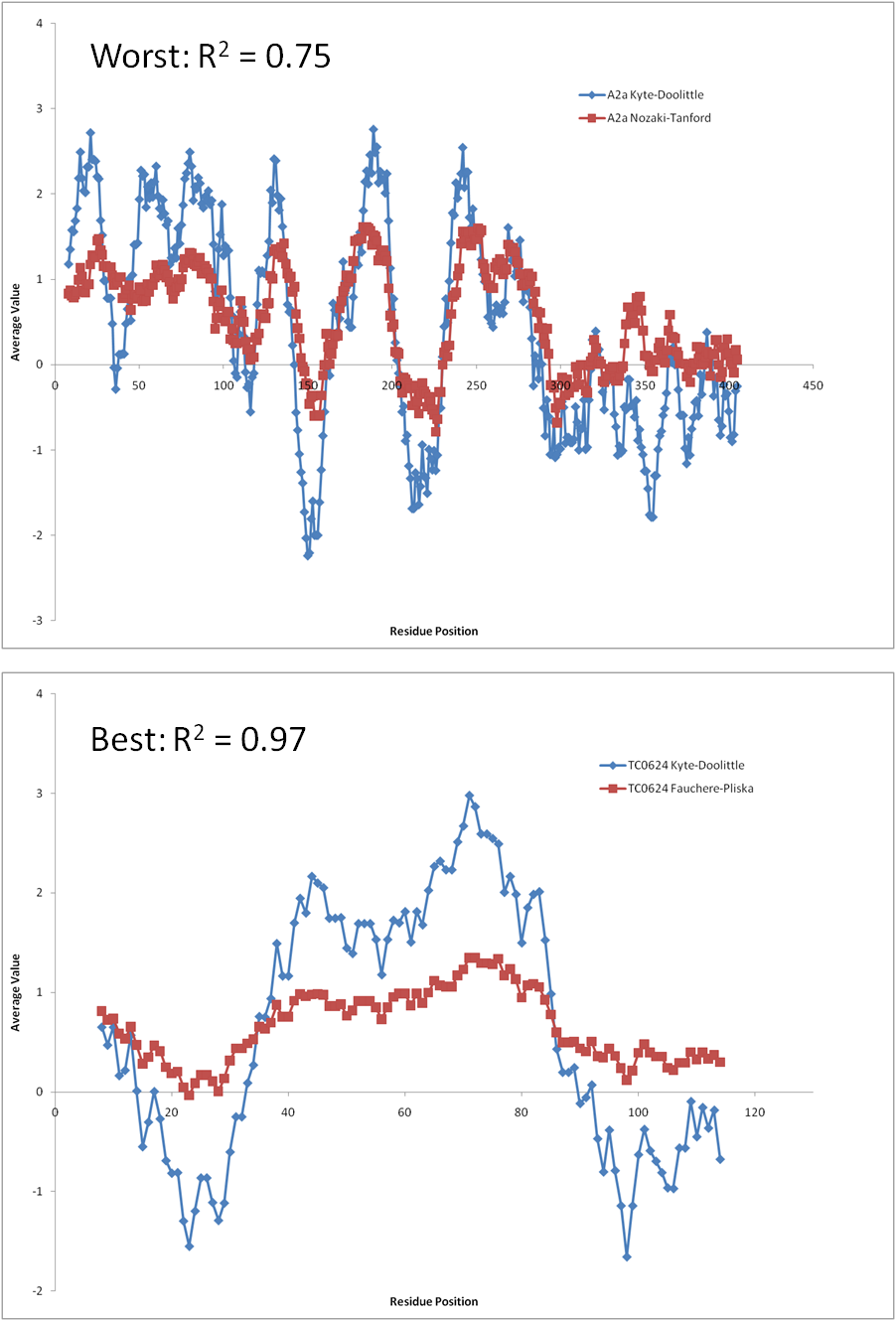


Next, the optimal *HePCaT* matches described in the main text were recomputed using the different profiles. Importantly, all *HePCaT* parameters were unchanged in these calculations except for the scale, and the calculations were executed on the public site at http://www.best.bio.jhu.edu/HePCaT. In all cases, the results indicated that the identical regions matched using the Kyte-Doolittle scale were also matched when different scales were used (Figure S2). The identical regions were matched regardless of the strength of the correlation of the aligned positions (Table S3). Table S3 shows that, although the Kyte-Doolittle scale happens to exhibit the nominally most similar matches, other scales, such as Rose, *et al.* and Radzicka-Wolfenden, approach that quality. These results are interpreted as evidence for the robustness of location and quality of optimal *HePCaT* matches, with respect to the exact details of hydrophobicity scale used.

**Figure S2. Examples of best and worst optimal matches using different hydrophobicity scales demonstrate that these matches are independent of scale.**

**
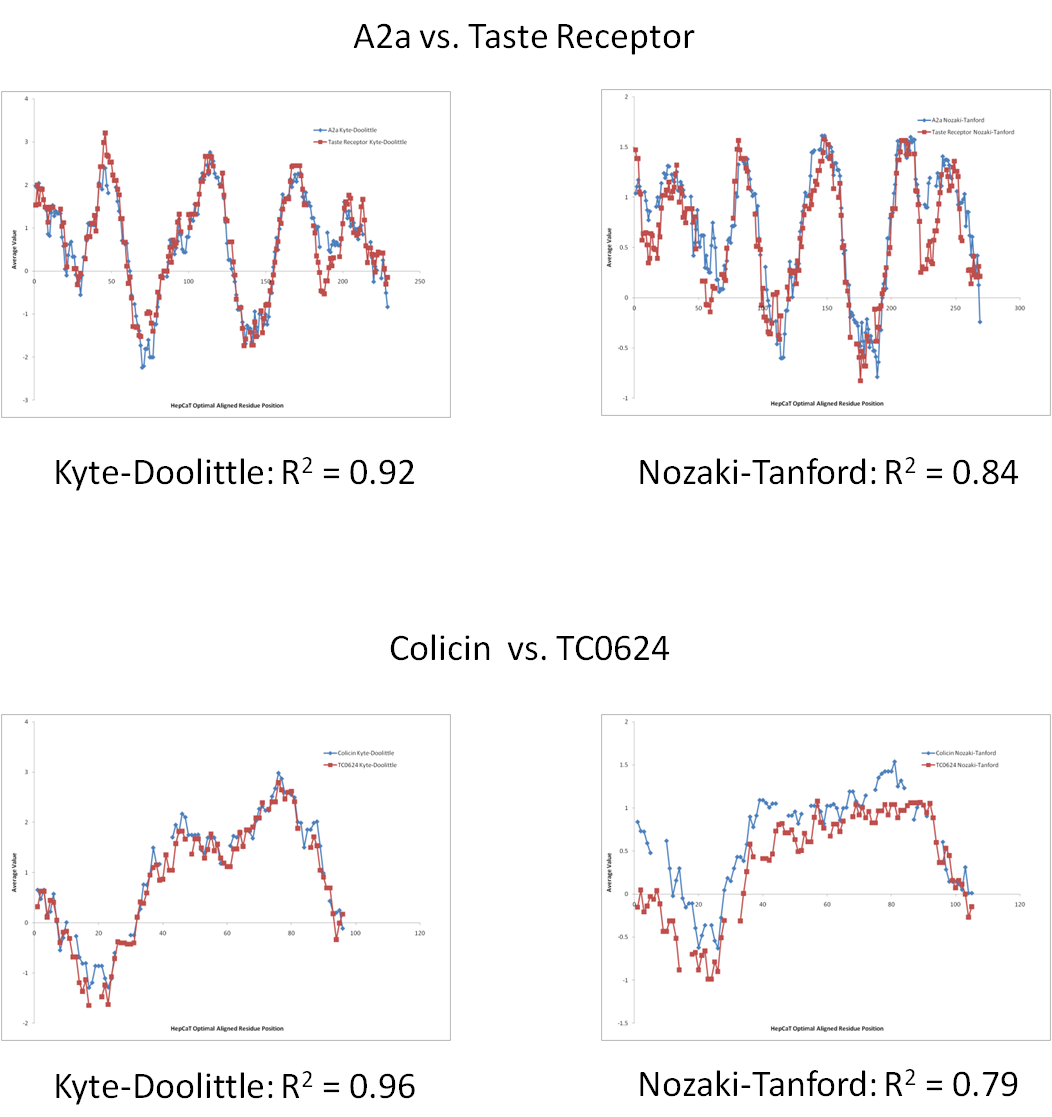
**

**Table S3. Pearson correlation coefficients (R^2^) between *HePCaT* optimally aligned positions using different hydrophobicity scales.**

| Protein Pair | Radzicka-Wolfenden | Nozaki-Tanford | Fauchere-Pliska | Rose, *et al.* | Kyte-Doolittle |
| --- | --- | --- | --- | --- | --- |
| Human A2a Receptor  *vs.*  Human Taste Receptor Type 2 Member 19 | 0.86 | 0.84 | 0.85 | 0.87 | 0.92 |
| *E. coli* Colicin  *vs.*  ORFan TC0624 | 0.92 | 0.79 | 0.90 | 0.93 | 0.96 |

**References**

1. Kyte J, Doolittle RF (1982) A simple method for displaying the hydropathic character of a protein. Journal of Molecular Biology 157: 105-132.

2. Andreeva A, Howorth D, Chandonia JM, Brenner SE, Hubbard TJ, et al. (2008) Data growth and its impact on the SCOP database: new developments. Nucleic Acids Res 36: D419-425.

3. Yomtovian I, Teerakulkittipong N, Lee B, Moult J, Unger R (2010) Composiiton bias and the origin of ORFan genes. Bioinformatics 26: 996-999.

4. Tomii K, Kanehisa M (1996) Analysis of amino acid indices for prediction of protein structure and function. Protein Engineering 9: 27-36.

5. Radzicka A, Wolfenden R (1988) Comparing the polarities of the amino acids: side-chain distribution coefficients between the vapor phase , cyclohexane, 1-octanol, and neutral aqueous solution. Biochemistry 27: 1664-1670.

6. Nozaki Y, Tanford C (1971) The solubility of amino acids and two glycine peptides in aqueous ethanol and dioxane solutions. Establishment of a hydrophobicity scale. Journal of Biological Chemistry 246.

7. Levitt M (1976) A simplified representation of protein conformations for rapid simulation of protein folding. Journal of Molecular Biology 104: 59-107.

8. Fauchere J, Pliska V (1983) Hydrophobic parameters pi of amino acid side chains from the partitioning of N-acteyl-amino-acid amides. European Journal of Medicinal Chemistry 18: 369-375.

9. Rose GD, Geselowitz AR, Lesser GJ, Lee RH, Zehfus MH (1985) Hydrophobicity of amino acids in globular proteins. Science 229: 834-838.

10. Creighton TL (1993) Proteins: Structures and Molecular Properties. New York: W.H. Freeman and Company.

11. Press WH, Teukolsky SA, Vetterling WT, Flannery BP (1992) Numerical recipes in C: the art of scientific computing. New York: Cambridge University Press.
